# Retraction: Ferrari Aggradi, CR, Falcier, E, Lizio, A, et al. Assessment of respiratory function and need for non‐invasive ventilation in a cohort of patients with myotonic dystrophy type 1 followed at one single expert centre. *The Clinical Respiratory Journal*. 2022; 1‐ 13. (https://doi.org/10.1111/crj.13516)

**DOI:** 10.1111/crj.13516

**Published:** 2022-06-27

## Abstract

The above article, published online on 27 June 2022 in Wiley Online Library (wileyonlinelibrary.com), has been retracted by agreement between the authors, the journal Editors in Chief, Professor Paul Jones and Professor Yuanlin Song and John Wiley and Sons Ltd. The retraction has been agreed due to an error with the publisher and author which caused a duplicate of the article to be published on 27 June 2022. The correct version of the article is to be found at: Carola R. Ferrari Aggradi, Elisa Falcier, Andrea Lizio, Alice Pirola, Jacopo Casiraghi, Alice Zanolini, Elena Carraro, Luca Mauro, Fabrizio Rao, Elisabetta Roma, Antonino Iannello, Elisa De Mattia, Andrea Barp, Sara Lupone, Valentina Gatti, Cristina Italiano, Valeria A. Sansone, “Assessment of Respiratory Function and Need for Noninvasive Ventilation in a Cohort of Patients with Myotonic Dystrophy Type 1 Followed at One Single Expert Center”, *Canadian Respiratory Journal*, vol. 2022, Article ID 2321909, 11 pages, 2022. https://doi.org/10.1155/2022/2321909.

